# Clinical Text Data in Machine Learning: Systematic Review

**DOI:** 10.2196/17984

**Published:** 2020-03-31

**Authors:** Irena Spasic, Goran Nenadic

**Affiliations:** 1 School of Computer Science and Informatics Cardiff University Cardiff United Kingdom; 2 Department of Computer Science University of Manchester Manchester United Kingdom

**Keywords:** natural language processing, machine learning, medical informatics, medical informatics applications

## Abstract

**Background:**

Clinical narratives represent the main form of communication within health care, providing a personalized account of patient history and assessments, and offering rich information for clinical decision making. Natural language processing (NLP) has repeatedly demonstrated its feasibility to unlock evidence buried in clinical narratives. Machine learning can facilitate rapid development of NLP tools by leveraging large amounts of text data.

**Objective:**

The main aim of this study was to provide systematic evidence on the properties of text data used to train machine learning approaches to clinical NLP. We also investigated the types of NLP tasks that have been supported by machine learning and how they can be applied in clinical practice.

**Methods:**

Our methodology was based on the guidelines for performing systematic reviews. In August 2018, we used PubMed, a multifaceted interface, to perform a literature search against MEDLINE. We identified 110 relevant studies and extracted information about text data used to support machine learning, NLP tasks supported, and their clinical applications. The data properties considered included their size, provenance, collection methods, annotation, and any relevant statistics.

**Results:**

The majority of datasets used to train machine learning models included only hundreds or thousands of documents. Only 10 studies used tens of thousands of documents, with a handful of studies utilizing more. Relatively small datasets were utilized for training even when much larger datasets were available. The main reason for such poor data utilization is the annotation bottleneck faced by supervised machine learning algorithms. Active learning was explored to iteratively sample a subset of data for manual annotation as a strategy for minimizing the annotation effort while maximizing the predictive performance of the model. Supervised learning was successfully used where clinical codes integrated with free-text notes into electronic health records were utilized as class labels. Similarly, distant supervision was used to utilize an existing knowledge base to automatically annotate raw text. Where manual annotation was unavoidable, crowdsourcing was explored, but it remains unsuitable because of the sensitive nature of data considered. Besides the small volume, training data were typically sourced from a small number of institutions, thus offering no hard evidence about the transferability of machine learning models. The majority of studies focused on text classification. Most commonly, the classification results were used to support phenotyping, prognosis, care improvement, resource management, and surveillance.

**Conclusions:**

We identified the data annotation bottleneck as one of the key obstacles to machine learning approaches in clinical NLP. Active learning and distant supervision were explored as a way of saving the annotation efforts. Future research in this field would benefit from alternatives such as data augmentation and transfer learning, or unsupervised learning, which do not require data annotation.

## Introduction

Clinical narratives represent the main form of communication within health care. In comparison with generically coded elements of electronic health records (EHRs), the narrative notes provide a more detailed and personalized account of patient history and assessments, offering a better context for clinical decision making [1]. Natural language processing (NLP) is a subfield of artificial intelligence that studies the ways in which the analysis and synthesis of information expressed in a natural language can be automated. It has repeatedly demonstrated its feasibility to unlock evidence buried in clinical narratives, making it available for large-scale analysis down the stream [2]. Traditionally, rule-based approaches were commonly used to unlock evidence of specific types [3]. Their development requires some form of direct interaction with clinical experts to convert their knowledge, often tacit, into a set of explicit pattern-matching rules.

Machine learning has long been hailed as a silver bullet solution for the knowledge elicitation bottleneck, the main argument being that the task of annotating the data manually is easier than that of eliciting the knowledge [4]. Nonetheless, the amount of data required to train a machine learning model may require as much time to annotate as the knowledge elicitation itself [5]. Much like the law of energy conservation, it seems that the knowledge required to inform the creation of an accurate computational model is simply transferred from one form to another. Instead of explicit knowledge in the form of rules, machine learning is based on implicit knowledge in the form of annotations and their distribution, with the time involved in their acquisition remaining virtually constant.

Another problem associated with the machine learning approach is the availability of clinical narratives given the sensitive nature of health data and privacy concerns [6]. These problems (ie, unavailability of manually annotated data) may result in the lack of representativeness of the training data and consequently substandard performance of the corresponding machine learning models. For these reasons, the main aim of this review was to provide systematic evidence on the properties of data used to train machine learning approaches to clinical NLP. In addition, we investigate the types of NLP tasks that have been supported by machine learning and how they can be applied in clinical practice.

The remainder of the paper is organized as follows. We start by explaining the methodology of this systematic review in detail. We then discuss the main findings of the review. Finally, we conclude the review by outlining future research directions in this field.

## Methods

### Overview

On the basis of the guidelines for performing systematic reviews described by Kitchenham [7], our methodology is structured around the following steps. First, research questions (RQs) were used to define the scope, depth, and the overall aim of the review. Next, a search strategy was designed to identify all studies that are relevant to the RQs in an efficient and reproducible manner. In addition, inclusion and exclusion criteria were defined to refine the scope. A critical appraisal of the included studies was conducted to ensure that the findings of the review are valid. During data extraction, the relevant information was identified from the included studies and semistructured to facilitate the synthesis of evidence and support the findings of the review.

### Research Questions

The overarching topic of this review is concerned with the properties of text data used to enable machine learning approaches to clinical NLP. The main aim of the review was to answer the RQs given in Table 1. RQ1 aims at describing the properties of data that are relevant for interpreting the performance of machine learning. These properties include size, provenance, heterogeneity, annotations, and others. Here, heterogeneity refers to content, structure, and clinical domains. RQ2 classifies the problems addressed by machine learning in the context of NLP into different types of computational tasks. Finally, RQ3 focuses on the ways in which NLP based on machine learning can be applied to tackle practical problems encountered in clinical practice.

**Table 1:** Research questions.

ID	RQ^a^
RQ1	What are the key properties of data used to train and evaluate machine learning models?
RQ2	What types of NLP^b^ tasks have been supported by machine learning?
RQ3	How can NLP based on machine learning be applied in clinical practice?

^a^RQ: research question.

^b^NLP: natural language processing.

### Search Strategy

We used PubMed as a search engine to retrieve relevant documents from the MEDLINE database of 28 million citations from life sciences and biomedical literature, which are indexed by Medical Subject Headings (MeSH). MeSH is a hierarchically organized controlled vocabulary used for manually indexing articles in MEDLINE in a uniform and consistent manner to facilitate their retrieval. We derived a list of search terms to describe the topic of this review: *machine learning, deep learning, text, natural language, clinical, health, health care,* and *patient*. Here, machine learning and deep learning are used to retrieve articles that employ this methodology. Note that MeSH includes the term *machine learning*, thus making it unnecessary to include specific machine learning techniques such as *support vector machines* or *conditional random fields* into the search query. The following 2 search terms, *text* and *natural language*, refer to the relevant type of input into the learning methods. The final 4 terms were used to refer to clinical applications. Owing to the broad nature and common use of the last 6 terms, their mentions were restricted to titles and abstracts only. In an attempt to prevent retrieval of nonoriginal studies and NLP applications developed to support systematic reviews, we negated the terms *literature*, *bibliometric*, and *systematic review*. Finally, to focus on the emerging application of machine learning, we restricted the search to the period from January 1, 2015. The search was performed on August 8, 2018. The search terms were combined into a PubMed query as follows:

((“machine learning”[All Fields] OR “deep learning”[All Fields]) AND (text[Title/Abstract] OR “natural language” [Title/Abstract]) AND (clinical[Title/Abstract] OR health [Title/Abstract] OR healthcare[Title/Abstract] OR patient [Title/Abstract]) NOT (literature[Title/Abstract] OR bibliometric [Title/Abstract] OR “systematic review”[Title/Abstract]) AND (“2015/01/01”[PDat] : “2018/08/08”[PDat])

We identified 389 candidate articles according to the described search strategy. The results were further screened against the selection criteria.

### Selection Criteria

The scope of this systematic review was formally defined by the inclusion and exclusion criteria given in Textboxes 1 and 2, respectively. Having screened the retrieved articles against the inclusion and exclusion criteria, a total of 149 articles were retained for further processing.

**Inclusion criteria.**

1. The study has to use natural language processing.
2. Machine learning has to be used to support such processing.
3. Input text has to be routinely collected within health care boundaries.
4. Input text has to be written or dictated.
5. The article has to be peer reviewed.
6. The full text has to be freely available online for academic use.

**Exclusion criteria.**

1. Articles written in a language other than English.
2. Natural language processing of a language other than English.
3. Natural language processing of spoken language.

**Figure 1:**
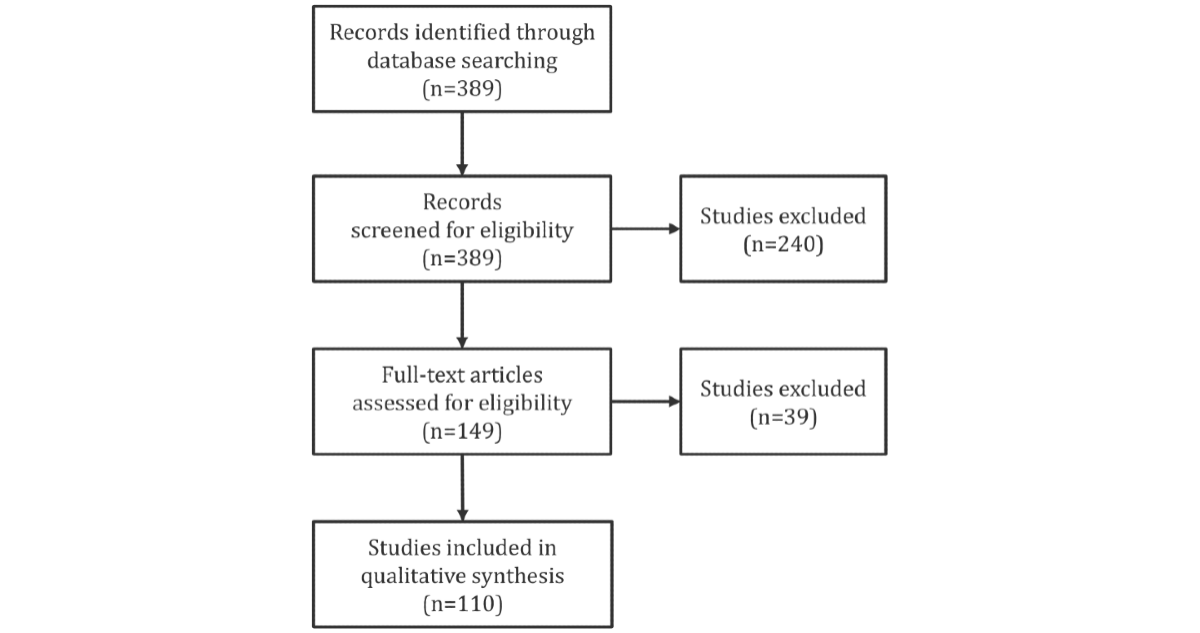
Flow diagram of the literature review process.

Given the interdisciplinary nature of articles considered for this review, we encountered a wide diversity of venues in which they were published. Not surprisingly, some studies put an emphasis on the clinical aspects but neglected to describe the computational aspects of the study in sufficient detail to support its reproducibility. To be included in this review, articles needed to provide sufficient information to support answering RQs defined in Table 1. In other words, they needed to describe the datasets used; define the NLP problem clearly; describe the features used to support NLP; state the machine learning methods used and, where appropriate, their parameters; and provide a formal evaluation of the results. A total of 39 studies were found not to match these criteria. This further reduced the number of selected articles to 110 [8-117]. Figure 1 summarizes the outcomes of the 4 major stages in the systematic literature review.

### Data Extraction

We explored the selected studies to extract data that contribute to answering the RQs given in Table 1. Data were extracted from the full text of articles under the following headings: data, task, clinical domain, and clinical application. The data properties considered included their size, provenance, collection methods, annotation, and any relevant statistics. The task was defined as a subfield of NLP (eg, text classification, information extraction (IE), named entity recognition (NER), and word sense disambiguation [WSD]). This was supplemented with task-specific information; for example, for NER, we also specified the type of named entities considered. Clinically relevant information was extracted to identify the potential for practical applications. The extracted data were then used to facilitate a narrative synthesis of the main findings.

## Results

The first step in developing a machine learning model is to collect data relevant to the problem at hand. Ultimately, the model’s performance will depend on the properties of such a dataset. We summarized these properties, including data size, key data sources, training annotations, and types of clinical documents considered.

### Size

**Figure 2:**
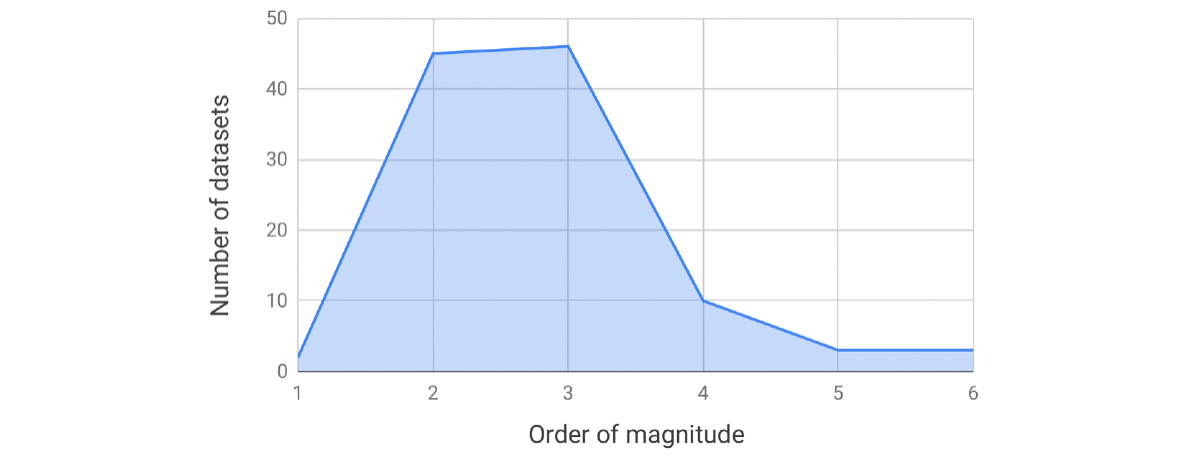
Distribution of data size on a logarithmic scale.

Among other factors, the performance of machine learning models and the significance of test results depend on the size of the dataset used for training and testing, respectively. In this section, we examine the size of datasets used in the studies included in this review. Owing to large variations in data sizes, we used a logarithmic scale to fit this information into the chart shown in Figure 2, which stratifies the datasets according to their order of magnitude. Some studies used as few as 40 documents [48] and as few as 15 patients [28]. The vast majority of datasets have the cardinality in the range of hundreds or thousands. Only 10 studies used tens of thousands of documents, with a handful of studies utilizing more than that despite the fact that machine learning approaches are data hungry in the sense that their performance is strongly correlated with the amount of training data available.

**Figure 3:**
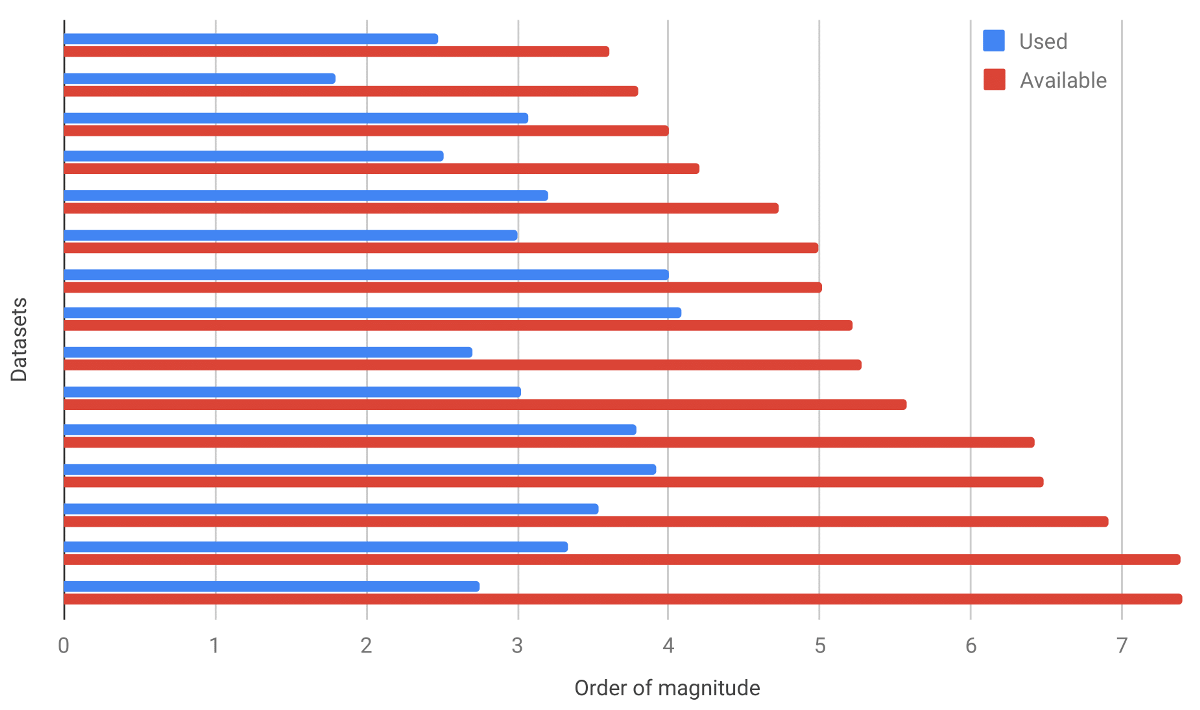
Data utilization on a logarithmic scale.

Relatively small datasets were utilized even when much larger datasets were available. Figure 3 demonstrates data utilization on a logarithmic scale, with some studies utilizing as little as 0.002% of available data [44] and as much as 11.88% [11]. Specific examples illustrate this issue: 500 from 188,843 [32], 300 from 4025 [59], 62 from 6343 [25], 323 from 16,000 [24], 1188 from 10,000 [11], 1610 from 52,746 [39], 1004 from 96,303 [112], 1058 from 376,487 [34], 10,000 from 103,564 sentences [36], less than 12,000 out of 137,522+28,159 [101], 562 from 2.5 million [44], 8288 from 2,977,739 [13], 6174 from 2.6 million [113], 3467 from 8,168,330 [68], and 2159 from 24 million [19].

### Annotation

The main reason for such poor data utilization is the annotation bottleneck faced by supervised machine learning algorithms, which require training data to be annotated to generalize them into predictive mathematical models. Compiling manually annotated corpora is both labor-intensive and error prone. The fact that annotations are task-specific means that the training data rarely get to be recycled. The labor and time limitations imposed on individual studies will naturally be correlated with the volume of manually annotated training data. Active learning aims to address the annotation bottleneck by involving human experts in the machine learning process in an attempt to improve performance with relatively small annotation effort [20,54,100]. An active learning algorithm can iteratively sample a subset of data for manual annotation, depending on the current predictive performance. Sampling strategies can be based on a disagreement between different predictive models or different measures of uncertainty, density, and expectation of a single predictive model. Such sampling depends on the quality of a predictive model and may not be efficient when retraining the model lasts relatively long. Alternatively, diversity measures can be used to prioritize annotation. For instance, pair-wise cosine similarity was used to compare sentences and prioritize those least similar to annotated sentences for annotation [20]. However, this may lead to the selection of outliers, whose presence in the training data can result in a degradation of predictive models. By considering representativeness and informativeness, outliers are less likely to be selected, thus leading to better coverage of the data characteristics and, consequently better predictive models. Here, the average similarity between a sentence and all other sentences indicates how representative it is [54]. The higher the similarity, the more representative the sentence is.

In principle, supervised learning approaches are convenient when labels are readily available. For instance, EHRs combine different types of data elements from unstructured data such as free text and images to structured data (ie, discrete elements such as numbers, dates, and codes) from controlled medical terminologies [118]. In the studies included in this systematic review, larger datasets (ie, those ranging from tens of thousands to millions, see Figure 2), were used mostly in cases where existing structured data were utilized as labels. For instance, in relation to hospitalization, readily available information about events such as in-hospital death [102], discharge [90], readmission [9], and emergency department visits [37] was used to train models to predict future events of this type well in advance to inform an appropriate course of action. Similarly, in relation to diagnostics, both prior (eg, imaging protocol [17,94]) and posterior (eg, test result [69]) information was utilized for supervision. International Classification of Diseases (ICD) diagnosis codes were used to train predictive models from historical data to identify patients at risk [16,22,50] or to facilitate disease surveillance [76]. Similarly, supervised models trained with ICD procedure codes otherwise used for billing can be used for cost optimization but also improving the quality of care [81]. Indeed, all of these examples have clear applications in care improvement and resource management. In some other cases (eg, classification of clinical notes into medical subdomains [103]), the utility of such information remains unclear.

Some types of learning problems such as WSD lend themselves well to semiautomated labeling based on greedy matching. Not surprisingly, the corresponding methods were tested on large datasets [33,105]. Similarly, using the concept of distant supervision, which utilizes an existing knowledge base to automatically annotate raw text, as much as 9.5 million clinical notes were annotated with adverse drug events [99]. Where manual annotation was unavoidable, crowdsourcing was explored. This approach is suitable for patient-facing problems such as readability of medical documents [116], where lay annotators are indeed ideally suited for the annotation task. The concept of crowdsourcing was explored for problems that require medical expertise [24]. Even though the interannotator agreement among crowdsourced workers was found to be much lower than that of medical experts, with Krippendorff alpha coefficient over .7, it was still found to be good agreement beyond chance. However, privacy constraints do undermine the feasibility of crowdsourcing in the context of clinical narratives.

### Provenance

Besides the small volume of training data, another issue that might affect the performance of machine learning methods trained on such data is their provenance. The structure and style of clinical narratives may vary greatly between institutions [119]. Therefore, when the provenance of data is confined to a small number of contributing institutions, the data may not be representative. This, in turn, may lead to overfitting, a modeling error that occurs when a complex model adapts to the idiosyncrasies of the training data and fails to generalize the underlying properties of the problem. Unfortunately, the majority of studies reviewed here were limited to the authors’ host institutions [8,10,12,15,17,22,24,25,28,30-33,35,40, 41,44,66,70,76,79,84-86,89,90,94,95,99,105,106,111,113]. Rarely are such datasets freely accessible to the community. A notable exception is the Medical Information Mart for Intensive Care (MIMIC) [120], a freely accessible critical care database that stores a wide range of clinical narratives, including radiology reports [87], clinical notes [102] and discharge summaries [16,39]. Although it is a single-site dataset, some consolation may be found in the sheer volume of data. More importantly, its public availability allows for rigorous and detailed direct comparison of competing approaches, a rare commodity in clinical NLP.

Only 9 studies used data from 2 institutions [36,47,50,56,61,100,103,109,112]. Three studies used data from 3 institutions [45,71,87]. A handful of studies managed to obtain data from multiple sources: 5 [38], 6 [73], 18 [19], and 28 [37]. The Veterans Health Administration (VHA) [121,122], as the largest integrated health care system in the United States, provides centralized access to data from multiple institutions, enhancing the credibility of results achieved on such data [13,14,29,34,55,68,72,77,97].

### Availability

Most datasets used in the included studies originated from a few institutions, thus offering no hard evidence about the transferability of machine learning models. Knowing that the format and style of clinical notes may vary substantially across institutions [119], it is not uncommon to observe a significant drop in performance when training a model in one institution and testing it in another [33,61,75,105,109]. This remains an ongoing concern for the clinical NLP community, where the confidentiality of data involved requires a careful balance between accessibility and privacy protection. In this section, we discuss wider availability of data that provide opportunities for secondary uses, including research. In this context, the NLP community challenges play an important role in providing access to clinical data to a wider pool of researchers and establishing benchmarks for future comparisons. Not surprisingly, many studies reviewed here have been enabled by the datasets shared in community challenges, which are described in Table 2.

**Table 2:** Datasets used in clinical natural language processing community challenges.

Dataset	Provenance	Documents	Size^a^	Annotations	Studies
2010 i2b2/VA [123]	PHC^b^, BIDMC^c^, UPMC^d^	Discharge summaries, progress reports	871	Medical problems, treatments, tests, and relations among them	[20,49,64,67,96,104]
2011 i2b2/VA [124]	PHC, BIDMC, UPMC, Mayo^e^	Discharge summaries, progress reports, radiology reports, pathology reports, other reports	978+164	Coreference chains for the problem, person, test, result, treatment, anatomical site, disease or syndrome, sign or symptom, etc	[63]
2012 i2b2 [125]	PHC, BIDMC	Discharge summaries	310	Clinical events, temporal expressions, temporal relations	[64]
2013 ShARe/CLEF eHealth [126]	BIDMC	Discharge summaries, electrocardiogram reports, echocardiogram reports, radiology reports	300	Disorders, acronyms, and abbreviations	[54,57,88,98,114]
2014 i2b2/UTHealth [127,128]	PHC	Longitudinal medical records	1304	Protected health information; risk factors for heart disease	[18,21,26,52,62,64,80,82,91,107,108]
2015 SemEval/THYME [129]	Mayo	Clinical notes, pathology reports	600	Times, events, and temporal relations among them	[60]
2016 CEGS N-GRID [130,131]	PHC	Psychiatric intake records	1000	Protected health information; symptom severity	[23,27,42,53,58,65,78,83,92]

^a^Size is expressed as the number of documents.

^b^Partners Health Care (PHC) is a nonprofit hospital and physician network that includes Brigham and Women’s Hospital and Massachusetts General Hospital.

^c^Beth Israel Deaconess Medical Center (BIDMC) is a teaching hospital of Harvard Medical School. Both organizations are based in Boston, Massachusetts, United States.

^d^The University of Pittsburgh Medical Center (UPMC) is a global nonprofit health enterprise that integrates over 35 hospitals, 600 clinical locations, and a health insurance division.

^e^The Mayo Clinic is a nonprofit academic medical center based in Rochester, Minnesota, which focuses on integrated clinical practice, education, and research. The clinic specializes in treating difficult cases through tertiary care.

Similarly, MIMIC dataset represents a key driver of open research in clinical NLP. It is notable for being the only freely accessible critical care database of its kind [120]. Data analysis is unrestricted once a data use agreement is accepted, enabling clinical research and education internationally. The open nature of the data supports the reproducibility of findings and enables continual research advances. MIMIC is a large, single-center database that stores deidentified, comprehensive clinical information relating to patients admitted to critical care units at the Beth Israel Deaconess Medical Centre in Boston, Massachusetts, United States, a large tertiary care hospital. Its content, which spans more than a decade, integrates different types of data (see Table 3). Of interest to this systematic review are free-text data, which include various types of notes and reports. Their integration with coded data offers an opportunity to circumvent manual annotation of data for supervised learning and evaluation purposes. For instance, Berndorfer and Henriksson [16] used a large dataset of 59,531 discharge summaries with at least one assigned ICD diagnosis code to automate the process of diagnosis coding. However, in many cases, accurate classification of medical conditions exists only in clinical narratives. Therefore, it may be necessary to annotate relevant phrases in the free text to train classification models. For instance, Gehrmann et al [39] manually annotated 1610 discharge summaries from MIMIC to automatically learn which phrases are relevant for 10 patient phenotypes considered. Similarly, Tahmasebi et al [87] manually annotated 860 radiology reports from MIMIC and 2 other institutions to evaluate an unsupervised approach to detecting and normalizing anatomical phrases.

**Table 3:** Description of clinical data types in the Medical Information Mart for Intensive Care.

Type	Description
Billing	Coded data recorded primarily for billing and administrative purposes.
Descriptive information	Demographic information, admission and discharge times, and dates of death.
Dictionaries	Look-up tables for cross-referencing identifiers (eg, codes) with associated definitions.
Interventions	Procedures such as dialysis, imaging studies, and placement of lines.
Laboratory measurements	Blood chemistry, hematology, urine analysis, and microbiology test results.
Medications	Administration records of intravenous medications and medication orders.
Notes	Free-text notes such as provider progress notes and hospital discharge summaries.
Physiologic information	Nurse-verified vital signs, approximately hourly (eg, heart rate, blood pressure, and respiratory rate).
Reports	Free-text reports of electrocardiogram and imaging studies (x-ray, computed tomography, ultrasound, and magnetic resonance imaging).

In addition to openness, an important driver of advancing state of the art in clinical NLP is an ability to access a wide range of data sources, many of which may not be compatible with national or organization-wide standards. As the largest integrated health care system in the United States, which provides care at 1243 health care facilities, including 172 medical centers and 1062 outpatient sites of care of varying complexity, the VHA [121,122] has the potential to address this challenge. The VHA offers veterans (ie, those who served in the active military, naval, or air service and who were discharged or released under conditions other than dishonorable) a wide range of inpatient, outpatient, mental health, rehabilitation, and long-term care services, which are all linked by an EHR platform. The construction of the VHA’s information infrastructure, the Veterans Information Systems Technology Architecture (VistA), began in 1982 and became operational in 1985. VistA integrates multiple applications seamlessly that are accessible via a graphical user interface, the Computerized Patient Record System, first launched in 1997. Designed primarily to support clinical care delivery rather than billing, the system has been used since 2004 to document all routine clinical activities currently storing more than 16 billion clinical entries.

On average, 1 million free-text notes (eg, progress notes and discharge summaries), 1.2 million provider-entered electronic orders, 2.8 million images (radiologic studies, electrocardiograms, and photographs), and 1 million vital signs were stored in VistA daily. Such proliferation of data quickly outgrew the original plans for storage capacity, network bandwidth, support staff, and information technology budget, leading to the construction of the Corporate Data Warehouse (CDW) in 2006. The new repository for patient-level data aggregated from across the VHA’s national health delivery system also hosts data from the legacy system, each featuring its own data rules, definitions, and structures. Given the slow process of normalizing these idiosyncrasies to a common standard and the rapidly increasing volume of data, the CDW allowed selective streaming of data from VistA and structuring them pragmatically in a way that minimizes redundancy. The CDW stores comprehensive patient-level data, which are used primarily to support health care delivery, but their unprecedented richness and volume provide a great opportunity for secondary uses such as quality improvement and research. To facilitate such uses, the VHA has partitioned a section of the CDW for use by health services and informatics investigators, who can access these data in secure workspaces within the VHA’s firewall. The VHA is developing mechanisms to fully deidentify data extracts so that they can be shared outside of the VHA.

Similar to MIMIC, integration of structured (coded) and unstructured (free-text) data offers an opportunity to circumvent manual annotation of data for supervised learning and evaluation purposes. In this manner, Ben-Ari et al [14] utilized postoperative notes of 32,636 patients by cross-referencing them to prescription data. However, most studies still rely on manual annotation of information that is not well documented in structured data. For example, Bates et al [13] manually annotated 8288 radiology reports as *fall* or *not fall* at the document level. Similarly, Maguen et al [68] annotated 3467 randomly selected psychotherapy notes with respect to the use of evidence-based psychotherapy. Patterson et al [77] manually annotated 2000 colonoscopy procedure notes with an indication, which included screening, nonscreening, noncolonoscopy, and unknown. Walsh et al [97] annotated 3900 snippets of text referring to axial spondyloarthritis in a corpus sampled from 500 million clinical notes and 120 million radiology notes. Divita et al [29] sampled 948 records from 164 preselected document types and annotated them manually to identify 5819 positively asserted symptoms within the documents. Kim et al [55] annotated a corpus of 1465 echocardiography reports, radiology reports, and other note types from multiple medical centers sampled at random for mentions and assessments of left ventricular ejection fraction. Fodeh et al [34] sampled 1058 clinical notes of 101 types and manually annotated fine-grained information about pain assessments, which included not only pain mention but also its features such as intensity, quality, site, and etiology. Meystre et al [72] sampled a cohort of 1083 patients and annotated their clinical notes of more than 10 preselected types with information regarding congestive heart failure treatment performance measures. These in-document annotations were summarized at the clinical note and patient level for binary classification of patients as meeting the treatment performance measure or not. These studies illustrate the extent of manual annotation effort involved in developing machine learning approaches to clinical NLP. Unfortunately, manual annotations remain underexploited because the fruits of such labor are rarely shared outside the original teams of investigators.

### Types of Narratives

The vast majority of studies focused on a single type of clinical narrative. This may be driven by a specific clinical application. For instance, Mai and Krauthammer [69] focused exclusively on free-text test orders to predict whether a patient would test positive for a particular virus in a quest to reduce viral testing volumes. To support service improvement, Elmessiry et al [30] focused solely on patient complaints. Similarly, applications related to patient safety focused on relevant documents such as adverse event reports [15], patient safety event reports [35], and incident reports [101].

Not surprisingly, most clinical applications of NLP focus on diagnosis and prognosis as they are central to medicine. Clinicians and health policymakers need to make predictions about the diagnosis and disease prognosis to support their decision making. These 2 applications focus primarily on various types of reports. For instance, electroencephalography reports were used to study epilepsy [41,70], whereas echocardiography reports were used to extract information relevant to cardiovascular medicine [55]. Most studies explored radiology reports [13,24,43,45,85,87,110,111]. They typically focus on a single imaging modality such as computer tomography [11,48,71,106,112] or magnetic resonance imaging (MRI) [17,47,94]. Such a segregated approach may be warranted by the intrinsic differences in the types of images produced, which may be reflected in the types of information discussed in the corresponding reports. For instance, MRI better differentiates between soft tissues than x-ray imaging does. Therefore, their respective reports may focus on different types of anatomical structures and their pathologies. This implies that machine learning models trained on one type of report may not be transferrable to another.

Nonetheless, aggregating findings from multiple imaging modalities [19,46,73] and other types of examination may increase diagnostic accuracy, especially when planning surgical treatments. In particular, pathology and radiology form the core of cancer diagnosis, leading to an initiative to integrate pathology and radiology studies to support making correct diagnoses and appropriate patient management and treatment decisions [132]. In this context, Bahl et al [10] combined mammographic reports, image-guided core needle biopsy reports, and surgical pathologic reports to avoid unnecessary surgical excisions. An important data source that supports this type of integration is RadBank**,** a database that links radiology and pathology reports [133]. It contains more than 2 million reports and allows full- text search by patient history, findings, and diagnosis by radiology and pathology. Still, the majority of studies focused on pathology reports alone [8,22,38,66,75,76]. Combinations of different report types were mostly used in enabling studies that focused on NLP tasks without a specific clinical application in mind (eg, NER approaches trained on electrocardiography, echocardiography, and radiology reports) [54,57,88,98,114].

Heterogeneity across different types of reports, including cardiac catheterization procedure reports, coronary angiographic reports together with integrated reports that combine history and physical report, discharge summary, outpatient clinic notes, outpatient clinic letter, and inpatient discharge medication report retrieved from the Emory Cardiovascular Biobank [134] was utilized to train robust machine learning models [115]. Different subsets drawn from clinical notes, admission notes, discharge summaries, progress reports, radiology reports, allergy entries, and free-text medication orders are typically used to support fundamental NLP applications such as spell-checking [56]; coreference resolution [63]; WSD [100], including that of abbreviations [105]; and NER [20,64]. Finally, colonoscopy reports were used to explore the feasibility of NLP in a clinical setting [77,93].

Discharge summaries are used as the primary communication means between hospitals and primary care and, as such, are essential for ensuring patient safety and continuity of care. Their content and structure may vary greatly between institutions and clinicians [135]. Typical components include dates of admission and discharge, reason for hospitalization, significant findings from history and examination, significant laboratory findings, significant radiological findings, significant findings from other tests, list of procedures performed, procedure report findings, stress test report findings, pathology report findings, discharge diagnosis, condition at discharge, discharge medications, follow‐up issues, pending test results, and information provided to patients and family. Practically, discharge summaries may be viewed as amalgamations of different types of clinical narratives, some of which we discussed previously. Although this may make their processing more challenging, any algorithms trained on discharge summaries are more likely to be applicable across a wider range of clinical narratives. Discharge summaries tend to provide the most informative accounts of patient phenotypes and have been used to automate cohort selection [39]. This also makes them well suited for training and testing NER approaches [59,96,104], extraction of relationships between them [49,67], or predicting diagnoses [16].

Other types of clinical narratives considered include physician notes [84], progress notes [25,40,90], EHR notes [74,81,116], surgical notes [14,79], and emergency department notes [50,109]. Unspecified type of clinical notes [102] were used mostly for classification [9,12,31,61,86,95,103,113], WSD [33], and disambiguation and IE [36,51,99].

Psychiatric notes were used mainly in an NLP community challenge to extract protected health information and symptom severity [23,27,42,53,58,65,78,83,92]. These narratives are key enablers of mental health informatics as the fine-grained context of actionable information does not readily lend itself to predefined coding schemes. Other types of documents used to support mental health applications include psychotherapy notes [68], event and correspondence notes [32], progress notes [40], and those in general clinical context including admission notes and discharge summaries [117].

Longitudinal EHRs were mainly used in NLP community challenges [18,21,26,52,62,80,82,91,107,108]. In practical applications, cumulative patient profiles were used to predict frequent emergency department visits [37]. Longitudinal records consisting of encounter and clinical notes were used to determine whether a candidate problem is genuine or not [28]. Similarly, encounter notes were used to determine whether a specific dermatological problem was definite, probable, or negative [44].

### Clinical Applications

**Figure 4:**
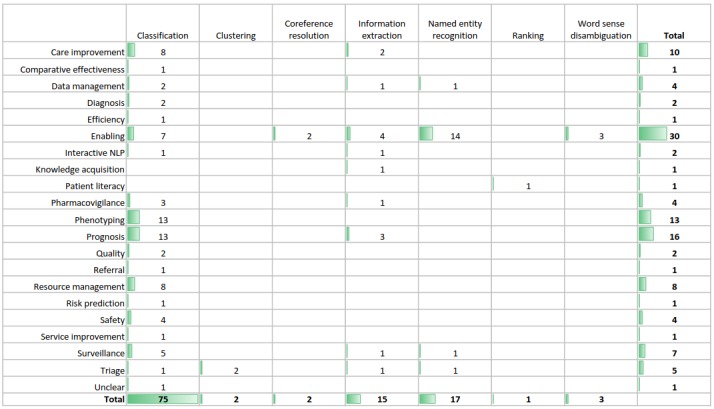
Clinical applications underpinned by natural language processing tasks.

This section focuses on the clinical applications of NLP approaches based on machine learning. We mapped 21 clinical applications against 7 NLP tasks (see Figure 4). It should be noted that we excluded a total of 39 studies that did not provide sufficient information to support answering RQs defined in Table 1. These studies may have described their own clinical applications, which are not discussed in this section.

Not surprisingly, the vast majority of studies focused on the task of text classification, which naturally lends itself to supervised machine learning. Most commonly, the classification results were used to support phenotyping, prognosis, care improvement, resource management, and surveillance.

EHR-based phenotyping approaches leverage data collected routinely in the course of health care delivery to identify cohorts of individuals that share certain clinical characteristics, events, and service patterns [136]. Their data can then be used for the secondary purposes of observational and interventional studies, prospective recruitment into clinical trials, health services research, public health surveillance, and comparative effectiveness research. Standardized computable phenotypes can enable large-scale studies while ensuring reliability and reproducibility. For instance, historical trial patient enrollment decisions were used to demonstrate the potential of NLP to increase trial screening efficiency by 450% and reduce workload associated with patient cohort identification by 90% [137]. Different types of events identified from EHRs include falls [13] and long bone fractures [43]. Most often, EHR phenotyping focused on a single medical condition, eg, axial spondyloarthritis [97], hypertension [89], systemic lupus erythematosus [95], dermatitis [44], obesity [61], celiac disease [22], epilepsy [41], autism [84], or psychiatric problems in general [40]. Two studies differentiated between multiple disorders. Tran and Kavuluru [92] focused on 11 mental disorders including attention-deficit hyperactivity disorder, anxiety, bipolar disorder, dementia, depression, eating disorder, grief, obsessive compulsive spectrum disorder, psychosis, and posttraumatic stress disorder. Gehrmann et al [39] focused on a less homogeneous list of 10 disorders including advanced cancer, advanced heart disease, advanced lung disease, chronic neurologic dystrophies, chronic pain, alcohol abuse, substance abuse, obesity, psychiatric disorders, and depression.

In terms of prognosis, text classification results were used to predict 3-month survival [12], the likelihood of intracranial hemorrhage [11] and the development of coronary artery disease [18,26,52,62,80,82,91,107,108] or prognosis based on cancer staging [75].

At the other end of the spectrum from text classification were lower-level tasks such as coreference resolution [63,110] and WSD [33,100,105], which were not associated with any particular clinical application. However, their importance lies in enabling other higher-level NLP tasks. Similarly, as a subtask of IE, NER can be used to support structuring text into predefined templates, whose slots need to be filled with named entities of relevant types. The majority of NER studies were related to NLP community challenges such as those described in studies by Uzuner et al [123], Suominen et al [126], and Stubbs et al [131]. They focused on entities such as medical problems, tests, and treatments [20,49,67,96,104]; disorders [54,57,88,98,114]; and protected health information [27,58,65].

Unlike NER, the more complex task of IE found a wider variety of clinical applications, the most prominent of which include prognosis and care improvement. For instance, cancer stage is one of the most important prognostic parameters in cancer, but this information is typically recorded in clinical narratives, which means that medical abstractors have to read through large volumes of text to extract such information. Given the importance and laboriousness of this task, it is not a coincidence that all IE approaches with prognosis as the most obvious clinical application focused on cancer staging [8,38,111]. Another IE approach related to cancer focused on extraction of symptoms experienced by patients during chemotherapy [36]. Rather than prognosis, this information can be used to improve patient care through modifying treatments and recognizing and managing symptoms. Similarly, extraction of information about assessments and medications can be used to improve management and outpatient treatment of patients suffering from chronic heart failure [72].

Triage is a process for sorting patients into groups based on their need for or likely benefit from medical treatment. Clustering, which is the task of grouping objects in a way that objects within a cluster are more similar to one another than to those in other clusters, can, therefore, naturally be applied to triage patients. Clustering was used to identify latent groups of lymphoma patients from their pathology reports [66]. Another study confirmed that automatically generated clusters of radiology reports coincided with major topics in radiology investigations [46]. Surprisingly, triage was not found to be a common clinical application of NLP and was largely associated with a single author [45-48].

### Summary

In this review, we examined the key properties of data used to train and evaluate machine learning models. We found that the size of the training dataset tends to be relatively small. For instance, the vast majority of studies included only hundreds or thousands of documents. Relatively small proportions were utilized for training even when much larger datasets were available. Beside their volume being small, training data were typically sourced from few institutions. In addition to the NLP community challenges such as i2b2, ShARe/CLEF eHealth, and CEGS N-GRID, most commonly used data sources were MIMIC and VHA. The vast majority of studies focused on a single type of clinical narratives, which ranged from imaging reports to hospital discharge summaries. Most often, training data were used to support the tasks of text classification, IE, and NER. Only a handful of studies focused on tasks such as clustering, ranking, coreference resolution, and WSD. Most commonly, the classification results were used to support clinical applications such as phenotyping, prognosis, care improvement, resource management, and surveillance. The remaining NLP tasks did not have clear clinical applications. In fact, the majority were used to enable other higher-level NLP tasks.

## Discussion

The use of text data in health informatics applications present quite a few challenges, the main ones being the preservation of patient privacy and the annotation bottleneck. Consequently, the training datasets become inflicted with problems typically associated with an unrepresentative sample. In other words, they may not reflect the distribution of characteristics of the target problem. In machine learning, such bias may lead to overfitting, a modeling error that occurs when a complex model adapts to idiosyncrasies of the training data and fails to generalize the underlying properties of the problem.

Unfortunately, most datasets used in the included studies originated from few institutions, thus offering no hard evidence about the generalizability and transferability of machine learning models. With the format and style of clinical notes varying substantially across institutions [119], a significant drop in performance was observed when training a model in one institution and testing it in another [33,61,75,105,109]. In this context, NLP community challenges play an important role in providing access to clinical data to a wider pool of researchers and establishing benchmarks for future comparisons. Not surprisingly, many studies included in this systematic review were enabled by the datasets shared in NLP community challenges. Unfortunately, relying on these challenges to provide clinical text data to NLP researchers seems like putting a Band-Aid on a proverbial bullet wound. Alternative opportunities have presented themselves in the form of synthetic health data, which contain the health records of realistic albeit not real patients. For instance, Synthea, the original open source synthetic health data software, can be used to simulate disease progression and the corresponding medical care to produce risk-free health care records at scale [138]. As synthetic data are not associated with any privacy concerns, crowdsourcing remains an option for their annotation though it may still require medical expertise, which remains an expensive commodity.

In terms of data annotation, lessons can be learned from other fields such as computer vision and speech processing, which have similarly been plagued by the lack of annotated data. They use data augmentation techniques to diversify data available for training of machine learning models without actually collecting any new data [139]. Similar techniques are now increasingly used to augment text data in a quest to improve generalization performance of the corresponding machine learning models [140-143]. Alternatively, transfer learning can be applied to leverage knowledge (features, parameters, etc) acquired in one domain and/or task with sufficient training data to support learning in another, which has got significantly less training data, thereby reducing expensive data annotation efforts [144,145]. In some cases, manual data annotation can be avoided altogether by applying the concept of distant supervision, which relies on an existing knowledge base to annotate text data automatically [146].

Some problems (eg, in-hospital death [102], discharge [90], readmission [9], and emergency department visits [37]), where labels are readily available, lend themselves naturally to supervised learning approaches. For instances, EHRs combine free-text data with codes from controlled medical terminologies, which can be utilized as class labels [118]. These codes were used to train predictive models from historical data to identify patients at risk [16,22,50], facilitate disease surveillance [76], or optimize the cost and quality of care [81]. For other problems, where data have to be annotated manually from scratch, insisting on supervised learning is very much like trying to fit a square peg through a round hole, leaving unsupervised approaches such as topic modeling largely underexplored even though they may be better fit for purpose for clinical applications such as EHR phenotyping, patient triage, care, and service improvement.

In summary, we identified the data annotation bottleneck as one of the key obstacles to machine learning approaches in clinical NLP. Active learning has been explored as a way of using the annotation efforts in a more strategic manner. However, the clinical NLP community could benefit from using alternatives such as data augmentation, transfer learning, and distant supervision. Ultimately, unsupervised learning avoids the need for data annotation altogether and, therefore, should be used more frequently to support clinical NLP.

## Abbreviations

CDW: Corporate Data Warehouse
EHR: electronic health record
ICD: International Classification of Diseases
IE: information extraction
MeSH: Medical Subject Headings
MIMIC: Medical Information Mart for Intensive Care
MRI: magnetic resonance imaging
NER: named entity recognition
NLP: natural language processing
RQs: research question
VHA: Veterans Health Administration
VistA: Veterans Information Systems Technology Architecture
WSD: word sense disambiguation
